# *In Vivo* Histamine Optical Nanosensors

**DOI:** 10.3390/s120911922

**Published:** 2012-08-29

**Authors:** Kevin J. Cash, Heather A. Clark

**Affiliations:** Department of Pharmaceutical Sciences, Northeastern University, Boston, MA 02115, USA; E-Mail: k.cash@neu.edu

**Keywords:** nanosensor, optode, histamine, *in vivo*

## Abstract

In this communication we discuss the development of ionophore based nanosensors for the detection and monitoring of histamine levels *in vivo*. This approach is based on the use of an amine-reactive, broad spectrum ionophore which is capable of recognizing and binding to histamine. We pair this ionophore with our already established nanosensor platform, and demonstrate *in vitro* and *in vivo* monitoring of histamine levels. This approach enables capturing rapid kinetics of histamine after injection, which are more difficult to measure with standard approaches such as blood sampling, especially on small research models. The coupling together of *in vivo* nanosensors with ionophores such as nonactin provide a way to generate nanosensors for novel targets without the difficult process of designing and synthesizing novel ionophores.

## 1. Introduction

*In vivo* detection and tracking of analytes is central for monitoring specific diseases, as well as a tool to advance knowledge about disease progression, personalized medicine, and biomarker discovery. Current approaches for *in vivo* analyte monitoring rely heavily on sampling techniques followed by offline analysis. Sampling techniques such as microdialysis [1,2] and blood sampling [3], while effective, have several key limitations including limited temporal resolution and the need to couple the technique with analysis methods such as HPLC or immunoassays. Both HPLC and immunoassays are inherently batch procedures, which prevent the use of these methods in continuous monitoring. Driven by the need for continuous glucose monitoring in diabetes, research groups and corporations have developed implanted enzymatic electrodes to enable continuous *in vivo* monitoring [4]. Unfortunately, these invasive approaches have drawbacks resulting from foreign body responses [4,5], leading to limited implantation lifetimes, as well as the difficulties of weekly re-implantation and lack of extension to other analytes.

To circumvent many of these limitations, we previously demonstrated the use of ionophore-based nanosensors for *in vivo* monitoring of sodium [6,7]. These spherical nanosensors are made of a highly plasticized hydrophobic polymer core with a biocompatible hydrophilic coating. The basic operation principle of these nanosensors is the same as that of the optodes on which they are based, and is explained in detail in other reports [8,9]. Briefly, a lipophilic ionophore binds to the target ion and extracts it into a polymer core. Also present in the polymer core is a pH sensitive dye (chromoionophore), which deprotonates to keep charge balanced in the core, and undergoes a shift in optical properties as a result. This transduction mechanism converts ionophore binding into a readily measured fluorescence change. However, one of the key limitations for this approach is the need to generate novel ionophores for each new desired target. Although ionophores exist for many atomic ionic species and several molecular ions [10], the ability to generate ionophores for larger molecules is difficult and precludes this approach for many desired analytes without significant additional research.

In this communication, we discuss the development of nanosensors utilizing a broadly amine-responsive ionophore that can be used to generate a sensor system to detect targets for which there is no available ionophore. This has been seen on macro-scale optodes before [11] as well as in other sensor architectures such as molecular beacons [12], but in this communication we develop an optode-based nanosensor for the detection of histamine. As our choice of ionophore we utilize the ammonium ionophore nonactin which has been thoroughly characterized in optodes by other groups [13,14]. In addition to recognizing ammonium, nonactin can bind to a range of amine containing small molecules [11]. The sensor mechanism is the same as other ionophore sensors where nonactin extracts histamine from the buffer into the polymer, which alters the fluorescence of the embedded pH sensitive fluorophore. This approach is based on our laboratory's significant experience in the design and application of this class of ion sensors (predominantly used for sodium sensing).

## 2. Experimental Section

### 2.1. Materials

Poly(vinyl chloride) (PVC), bis(2-ethylhexyl) sebacate (DOS), tetrahydrofuran (THF), 4-(2-hydroxyethyl)piperazine-1-ethanesulfonic acid (HEPES), dichloromethane, 9-dimethylamino-5-[4-(16-butyl-2,14-dioxo-3,15-dioxaeicosyl)phenylimino]benzo[a]phenoxazine (chromoionophore II; CHII), 9-(diethylamino)-5-[(2-octyldecyl)imino]benzo[a]phenoxazine (chromoionophore III; CHIII), nonactin (ammonium ionophore I), sodium tetrakis[3,5-bis(trifluoromethyl)phenyl] borate (NaTFPB), potassium tetrakis[3,5-bis(trifluoromethyl)phenyl] borate (KTFPB), and histamine dihydrochloride were purchased from Sigma Aldrich (St. Louis, MO, USA). 1,2-disteroyl-sn-glycero-3-phosphoethanolamine-N-[methoxy(polyethylene glycol)-550] ammonium salt in chloroform (PEG-lipid) was purchased from Avanti Polar Lipids (Alabaster, AL, USA). Phosphate buffered saline (PBS, pH = 7.4) was purchased from Life Technologies (Grand Island, NY, USA).

### 2.2. Optode and Nanosensor Fabrication

Protocols used in this report are based on those previously described [7,15]. In brief, the process of fabricating optodes and nanosensors starts with formulation of an optode cocktail comprising 500 μL of THF containing PVC, DOS, and the sensing components. These components include an ionophore, in this work nonactin, a chromoionophore, in this work both chromoionophore II (CHII) and chromoionophore III (CHIII) were used, and an ionic additive, NaTFPB or KTFPB. The ratio of these components is tuned to control the response of the nanosensors. The initial CH III optodes and nanosensors utilize a ratio of 20:40:1.5:0.1:0.05 PVC:DOS:nonactin:CHIII:KTFPB (mg/cocktail). CHII based nanosensors utilize a ratio of 30:60:3:0.5:10 PVC:DOS:nonactin:CHII:NaTFPB (mg/cocktail) for initial *in vitro* research and a ratio of 30:60:3:0.5:7.5 for *in vivo* work. Optode membranes are prepared by spotting 2 μL of CHIII based optode cocktail onto a 5 mm glass coverslip at the bottom of a 96 well plate. The THF evaporates to leave behind the optode membrane.

Histamine nanosensors were fabricated using methods previously reported for ion sensitive nanosensors [7,15]. In a scintillation vial, 2 mg of PEG-lipid was dried and then resuspended in 5 mL PBS with a probe tip sonicator for 30 seconds at 20% intensity (Branson, Danbury, CT, USA). Fifty μL of the optode cocktail was combined with 50 μL of dichloromethane, and added to the PBS/PEG-lipid solution under probe tip sonication (3 minutes, 20% intensity). The nanosensor solution was filtered with a 0.22 μm syringe filter to remove excess polymer (Pall Corporation, Port Washington, NY, USA). Nanosensors were sized using dynamic light scattering (DLS) with a Brookhaven 90Plus (Brookhaven Instruments, Holtsville, NY, USA).

### 2.3. *In Vitro* Characterization

#### 2.3.1. Optode Characterization

Histamine optodes were prepared on the bottom of a 96 well plate as described above. Three hundred μL of 10 mM HEPES buffer pH 7.4 was added to each well and allowed to equilibrate overnight (14 hours) to hydrate the optodes. The buffer was replaced with 200 μL of fresh buffer, and optode fluorescence was monitored using a SpectraMax M3 plate reader (Molecular Devices, Sunnyvale, CA, USA) with excitation at 635 nm, emission at 680 nm and a cutoff filter at 665 nm. After 1.5 hours this buffer was replaced with a histamine solution in HEPES (0 mM, 1 mM, 10 mM or 100 mM) and fluorescence intensity was monitored for 1.5 hours. The histamine solution was replaced with fresh buffer to regenerate the optodes and monitored for 1.5 hours. Another two cycles (1.5 hours histamine solution, 1.5 hours buffer) was performed to assess reversibility and reuse of the optodes. Fluorescence data was normalized by dividing by the fluorescence of the final point in the final cycle, and plotted as normalized fluorescence.

#### 2.3.2. Nanosensor Characterization

The same optode cocktail used for optode characterization was fabricated into nanosensors using the procedure outlined above. Nanosensors were added to a 96 well plate and scanned in the absence of histamine using the same wavelengths as for optode data acquisition. Histamine solutions were then added to the wells to final concentrations of 0 mM through 22 mM and the plate was rescanned. Data was normalized well-by-well to intensity before addition followed by normalization to intensity at 0 mM histamine. Nanosensors with chromoionophore II as the indicator were fabricated and characterized in a similar fashion. Optical wavelengths used for absorbance spectrums of CHII nanosensors were between 400 and 800 nm, 5 nm/step, and, for absorbance, endpoint measurements were made at 515 nm, 660 nm, and 570 nm (see supplementary information for absorbance results). Fluorescence was measured with excitation at 660 nm, emission at 700 nm with a cutoff filter at 695 nm. Fluorescence spectra were obtained with excitation at 660 nm, emission from 680–800 nm with a 2 nm step and a cutoff filter at 665 nm. Histamine concentrations used for measurement ranged from 10 nM to 50 mM and data was normalized to a 0 mM solution. The data for the calibration curve was fit to a Hill equation using Origin 8 software (OriginLab, Northampton, MA, USA) in order to determine the K_d_ of the response.

Chromoionophore II nanosensors were also calibrated *in vitro* utilizing a Lumina II *in vivo* imaging system (Caliper Life Sciences, Hopkinton, MA, USA). Nanosensors were combined with histamine solutions in a 96 well plate (100 μL final volume, blank and 100 nM to 50 mM histamine concentrations). This plate was imaged with high lamp power, excitation filter centered at 640 nm (30 nm bandpass), emission filter from 695 nm to 770 nm, and a 1 second exposure. For data analysis regions of interest were drawn over each well using Living Image 4 software (Caliper Life Sciences) and total fluorescent intensity values were obtained for each well and normalized to data for 0 mM histamine and fit as above.

### 2.4. *In Vivo* Studies

All *in vivo* studies were approved by the institutional animal care and usage committee (IACUC) of Northeastern University as well as the US Army Medical Research and Materiel Command (USAMRMC) Animal Care and Use Review Office (ACURO). The mice used in this research were male CD-1 Nude mice from Charles River (Wilmington, MA, USA). All experiments were carried out at Northeastern University.

Imaging experiments were conducted using a Lumina II *in vivo* imaging system (IVIS). The IVIS was used in fluorescence mode with high lamp power, excitation filter centered at 640 nm (30 nm bandpass), emission at 700 nm (20 nm bandpass), 1 second exposure, and images taken every minute. Nanosensors were concentrated approximately 10X for injection by using Amicon Ultra centrifugal filters (10 kDa cutoff). For data analysis of each experiment, a region of interest encompassing the injection area was selected and total fluorescent intensity was recorded. Each intensity value was normalized to the same spot at the first time point after injection of histamine (see Supplementary Figure S4 for an example of the normalization process). Normalizing the data to the first time point results in an increase in error bars as time increases due to slight differences between the three nanosensor injections. All animals were sacrificed after experiments were completed.

The first *in vivo* experiment was a coinjection of histamine and nanosensors to observe nanosensor reversibility. One mouse was anesthetized with isoflurane and given three subcutaneous injections along the left side of the back of 30 μL each of nanosensors diluted with PBS. Along the right side of the back the mouse was given three injections of 30 μL each of nanosensors diluted with histamine in PBS to a concentration of 80 mM. After injections, the mouse was imaged every minute for 90 minutes total. Two injections, one from each batch, were too close together to separate fluorescence for each one, and they were discarded for data processing resulting in two nanosensor injections for each curve in the coinjection data.

Following *in vivo* experiments involved intraperitoneal (i.p.) administration of histamine. For each experiment two mice were anesthetized with isoflurane and given three subcutaneous injections along the centerline of the back of 30 μL of nanosensors. Mice were imaged for 20 minutes to establish a baseline followed by an i.p. injection of histamine (75 mg/kg in PBS) or PBS alone (matched volume). Mice were then imaged for approximately 90 minutes following histamine administration. This experiment was repeated for a total of eight mice (four experimental, four control).

## 3. Results and Discussion

### 3.1. *In Vitro* Characterization

**Figure 1.**
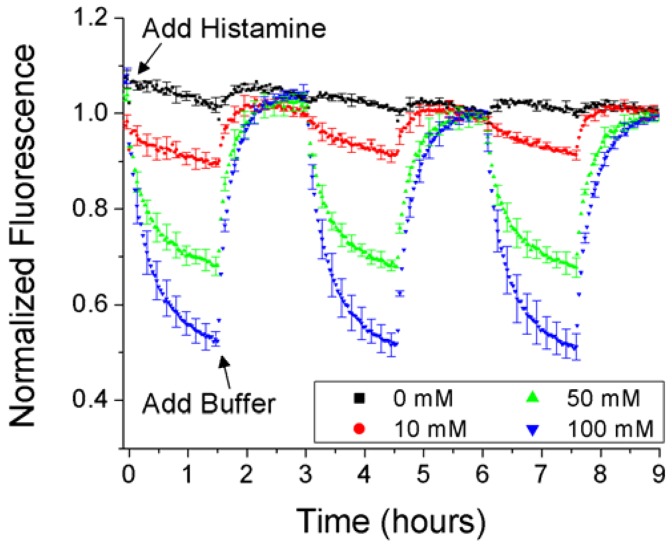
Bulk optodes utilizing nonactin as an ionophore are able to reversibly detect histamine in aqueous solutions. Note: to clarify image, only one of every five error bars is shown here. Please see Supplementary Information for the full dataset.

Development of a nanosensor approach for the detection of histamine begins with bulk optode characterization of the formulation. This approach allows for simple formulation changes as well as the ability to assess the reversibility of the sensor system. For continuous *in vivo* monitoring, reversibility is a key attribute enabling sensors to sense decreases in analyte concentrations during monitoring. The bulk optode, approximately 5 mm in diameter, is prepared on a glass disk in the bottom of a well plate. The optode is initially soaked in buffer, and then histamine is added to the wells with different concentrations in different wells. Replacing the histamine solution with fresh buffer results in a regeneration of the sensor back to the initial signal. Histamine optodes show a clear dose-dependent response as well as good reversibility for three cycles of histamine addition (Figure 1). The slow kinetic response is a result of the large scale of the optode relative to nanosensors, which have been shown to have at least millisecond response times [16].

**Figure 2.**
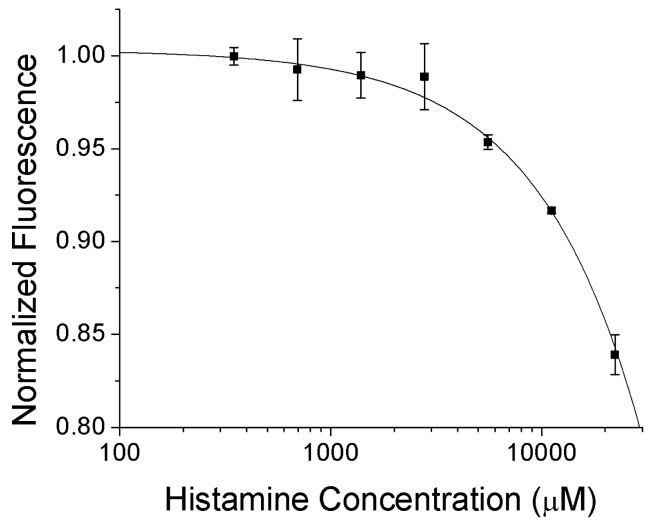
Histamine sensitive nanosensors show a clear dose response change in fluorescence with varying histamine concentrations.

As initial testing with the bulk optode showed reversible response to histamine, we synthesized nanosensors using the same optode cocktail formulation and well established fabrication procedures [7]. The resulting nanosensors were 130 nm in diameter with a polydispersity index of 0.2 (by DLS), similar to previous sensors developed by our group which have been more thoroughly characterized [15]. These nanosensors respond to histamine in a dose dependent manner (Figure 2) with a K_d_ of 125 mM. Additionally, the fluorescence of the nanosensors does not change in response to additions of urea, which is promising for *in vivo* application (data not shown).

**Figure 3.**
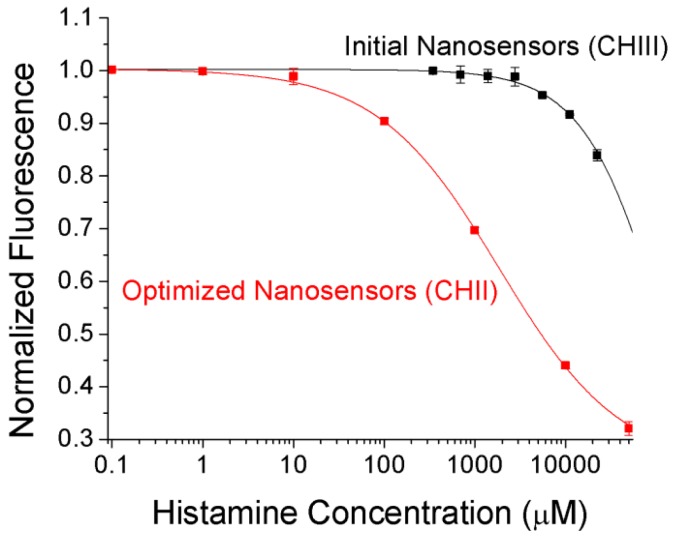
Chromoionophore II enables detection of histamine at a significantly lower concentration (K_d_ = 1.9 mM) than earlier work with Chromoionophore III (K_d_ = 125 mM) because of the lower pK_a_ of the indicator.

**Figure 4.**
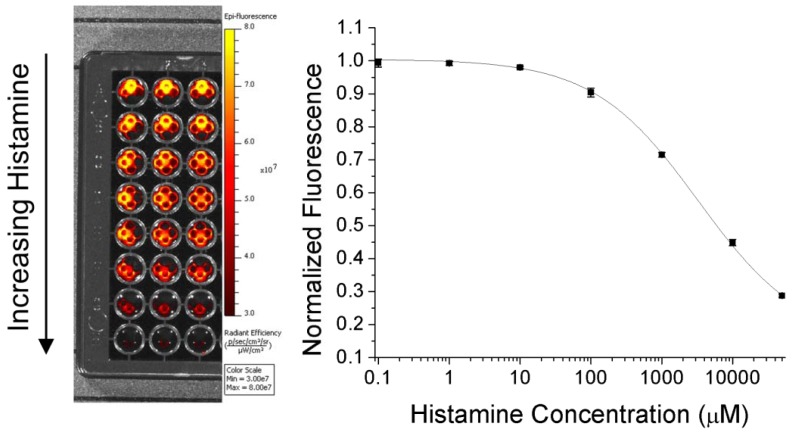
IVIS animal imager calibration of histamine nanosensor response. The well plate calibration, with histamine concentration increasing down the plate (left), responds to histamine (right) in the same manner as that obtained in the plate reader.

Although these initial tests were promising, the K_d_ and effective detection limits were too high to be of use for physiological monitoring. In optode-based sensors, the key driving factor for the response range is the choice of chromoionophore. In order to shift the binding curve lower, we reformulated the sensors to replace Chromoionophore III which was used for initial tests with Chromoionophore II for remaining research. This chromoionophore has a significantly lower pK_a_ (10.2 *vs.* 13.4 for CHIII [9]) which means that the optical properties of the nanosensors will shift at lower histamine concentrations. This reformulation improves the K_d_ of the nanosensors from 125 mM to 1.9 mM (Figure 3). While high compared with physiological plasma histamine concentrations (up to 8 μM after immune system stimulation [2,17]), the K_d_ is significantly lower than the histamine concentration in mast cells (100 to 500 mM [18]) which can lead to high local tissue concentrations upon mast cell release. The fluorescence of the chromoionophore itself, while not as intense as other fluorophores, is clearly visible in the IVIS animal imager (Figure 4), and has the same calibration as nanosensors in the plate reader, despite the vastly different optical setup.

### 3.2. *In Vivo* Characterization

**Figure 5.**
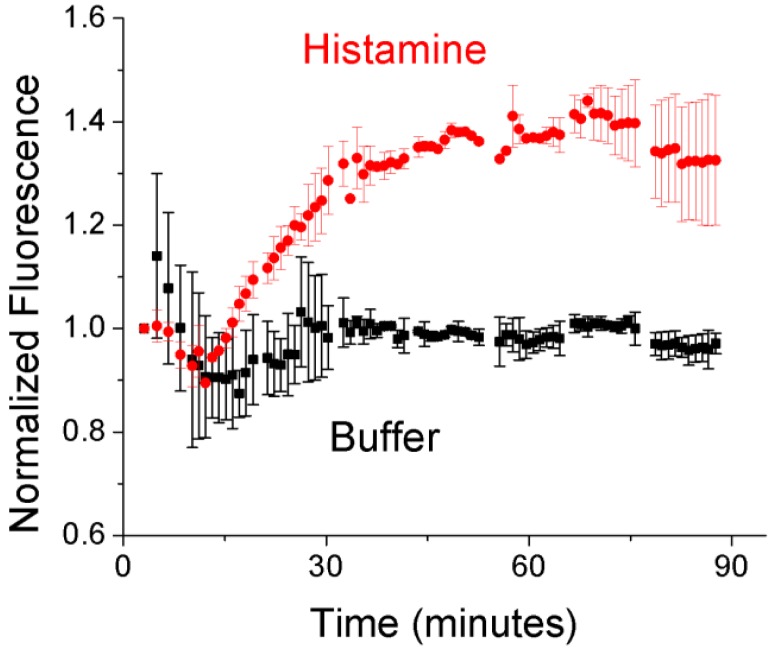
Nanosensors injected with histamine in the carrier solution show an increase in fluorescence as histamine is removed from the injection site. This demonstrated both *in vivo* specificity as well as nanosensor reversibility. Sensors injected with buffer show no signal change.

With *in vitro* demonstration of nanosensor response to histamine completed, we proceeded to *in vivo* proof of concept testing. Initial *in vivo* experiments consisted of subcutaneous injections along the backs of mice of either (a) histamine nanosensors in buffer or (b) histamine nanosensors with histamine in the carrier solution. After the injections were made, nanosensor fluorescence was tracked as the histamine diffused away from the injection site and was degraded. As histamine concentration and nanosensor fluorescence are inversely related, the removal of histamine led to an increase in fluorescence from the nanosensors injected with histamine and minimal change to those injected without histamine (Figure 5).

**Figure 6.**
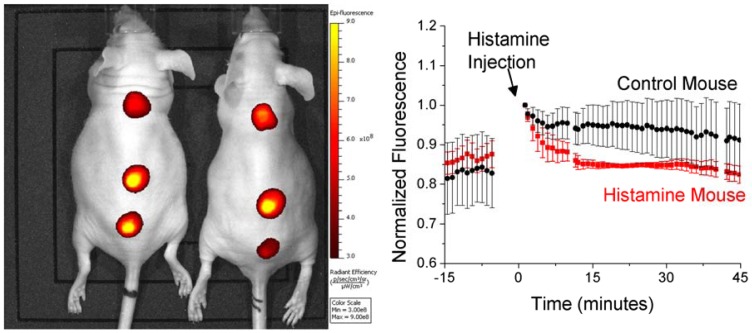
Histamine nanosensors can track systemic changes in histamine concentration. An example image (left) of two mice with histamine sensitive nanosensors implanted along their backs. One mouse is administered histamine while the other is given a control injection of buffer. Nanosensor fluorescence is tracked to monitor response to histamine (right).

In addition to monitoring local histamine concentrations, these nanosensors can be applied to track systemic histamine levels. In order to demonstrate this, we again injected nanosensors along the backs of mice, but altered systemic histamine levels via an i.p. injection of either histamine or buffer as a control (Figure 6). Nanosensor fluorescence is monitored for both the experimental and control mice and shows a rapid response to the rise in systemic histamine levels (Figure 6). As histamine levels increase, the nanosensor fluorescence drops when compared with the nanosensors in the control mouse. We repeated this experiment a total of four times (eight mice, four separate experiments) in order to gauge reproducibility of this approach for monitoring systemic histamine fluctuations (see supplementary information for related data). All of the experiments showed the same trend of a drop in fluorescence intensity after injection indicating histamine detection. The nanosensors are not yet capable of quantification of histamine concentrations *in vivo*, due to effects such as varying skin absorption. However, based on the signal change obtained the concentration detected is above the plasma concentration observed in physiological processes but below that of local changes near mast cell release. Future work will focus on the incorporation of reference signals to enable more quantitative measurements.

## 4. Conclusions/Outlook

In this communication we have demonstrated the fabrication and application of nanosensors for the *in vivo* detection and monitoring of histamine. We utilized a well-known ammonia ionophore, nonactin, as the recognition element of these nanosensors and demonstrated that the use of a broad spectrum ionophore such as this can generate nanosensors which function even in the analytically complicated *in vivo* environment. Despite the presumed lack of specificity that this ionophore may provide, these nanosensors were able to track histamine kinetics *in vivo* even at low analyte concentrations and a wide range of potential interferents. The use of enzymes coupled with this approach may be used to improve specificity in cases where it is necessary. This approach opens up the possibility of detecting a broad range of target analytes for which, like histamine, there is currently not an available ionophore recognition element. Future research should focus on two key areas in order to expand the utility of this approach. First, the range of ionophores which can be used in this manner should be extended. Until now, specificity has been a key focus of ionophore development, although this work highlights the advantage of a less specific ionophore in recognition of larger, more complicated, analytes. A second area of future research is to improve fluorescent signaling of the nanosensors through methods such as adding reference fluorophores or utilizing the inner filter effect [19,20] with brighter and more stable fluorophores than the chromoionophore, potentially increasing sensitivity. This will enable better transdermal imaging and as a result improve detection limits as well as measurement confidence. *In vivo* imaging of a wide range of analytically valuable targets will be made possible through development of nanosensors based on broadly reactive ionophores as recognition elements.

## Supplementary Material
