# Supplementary material for: In Vivo Histamine Optical Nanosensors
Source: Sensors (Basel). 2012 Aug 29;12(9):11922–32. doi: 10.3390/s120911922 (PMC3478817; doi:10.3390/s120911922)
Supplement: Supplementary file 1 [file sensors-12-11922-s001.pdf]

OPEN ACCESS
sensors
ISSN 1424-8220
www.mdpi.com/journal/sensors

Supplementary Information

In Vivo Histamine Optical Nanosensors. *Sensor* 2012, *12*, 11922-11932

Kevin J. Cash and Heather A. Clark *

Department of Pharmaceutical Sciences, Northeastern University, Boston, MA, USA;
E-Mail: k.cash@neu.edu

* Author to whom correspondence should be addressed; E-Mail: h.clark@neu.edu;
Tel.: +1-617-373-3091; Fax: +1-617-373-8886.

Supplementary Results

Figure S1. Dataset for Figure 1 with all error bars plotted.

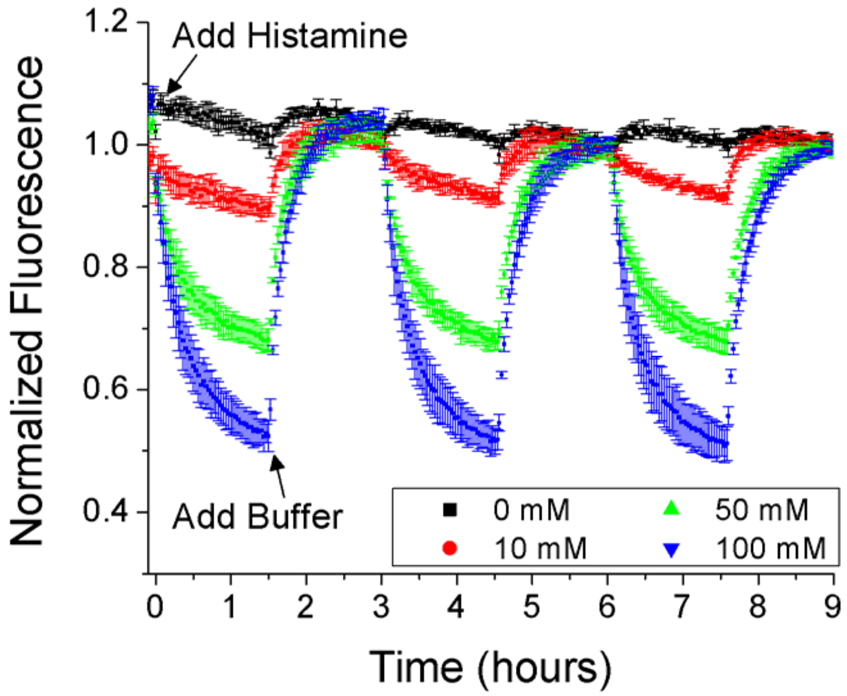

Figure S2. The absorbance characteristics of histamine nanosensors. The absorbance spectrum of Chromoionophore II (left) demonstrates a ratiometic change in response to histamine additions (0 mM, 10 nM to 50 mM). The ratio of the two absorbance peaks (right, 660 nm/515 nm) yields a dose response curve with a K_d of 1.3 mM. As fluorescence is used for *in vivo* experiments, it was more thoroughly investigated for *in vitro* calibration than absorbance.

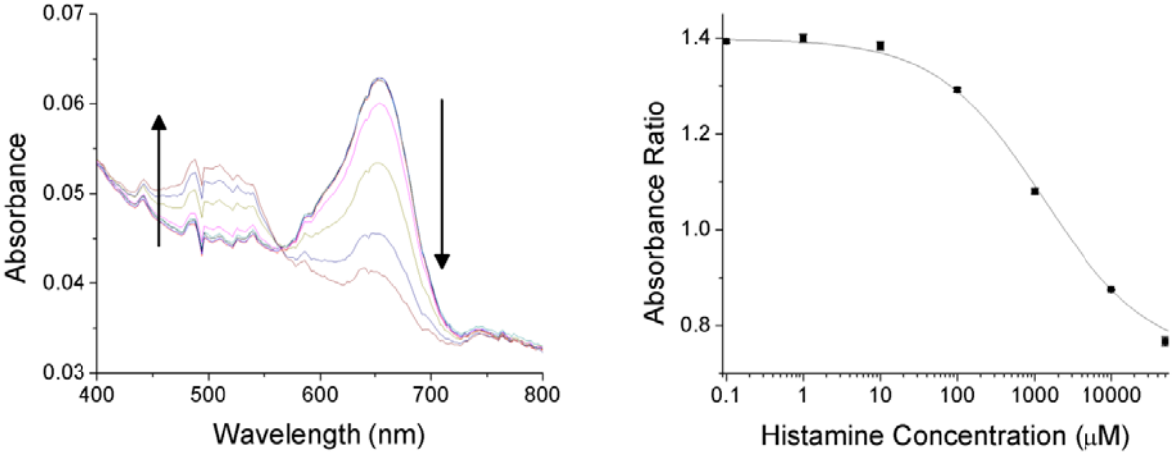

Figure S3. Fluorescence characteristics of histamine nanosensors. The fluorescence spectrum (left) and single point fluorescence measurements (right) for the nanosensors match the trend in absorbance data. The single point measurements on the right were normalized by the 0 μM data point to generate the Chromoionophore II data in Figure 3.

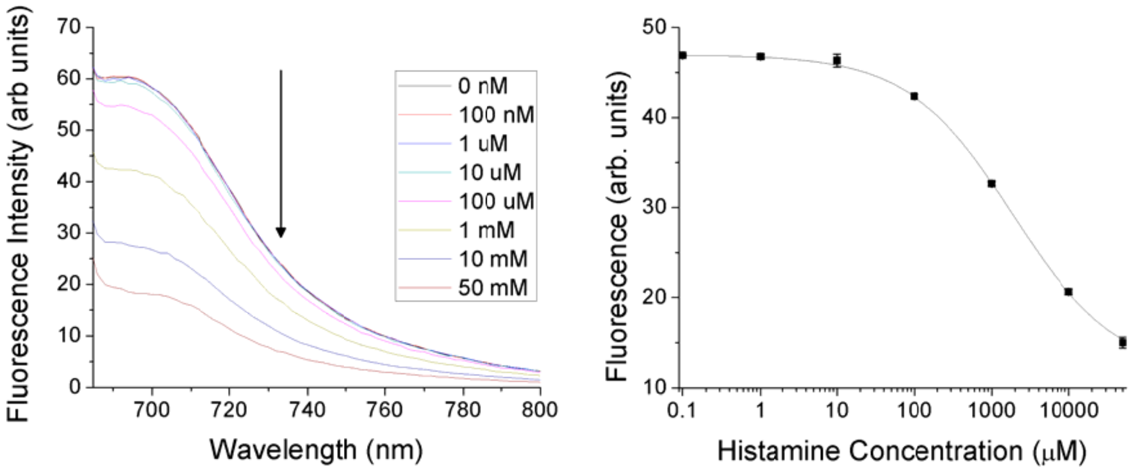

Figure S4. Raw fluorescence intensity data from the experiment in Figure 6 of the manuscript. The intensity (left) of each spot is normalized to the intensity after histamine injection in order to control for nanosensor injection depth, position on the animal, and other factors and then averaged together for each mouse. This results in the normalized data (right), which is also Figure 6 of the manuscript.

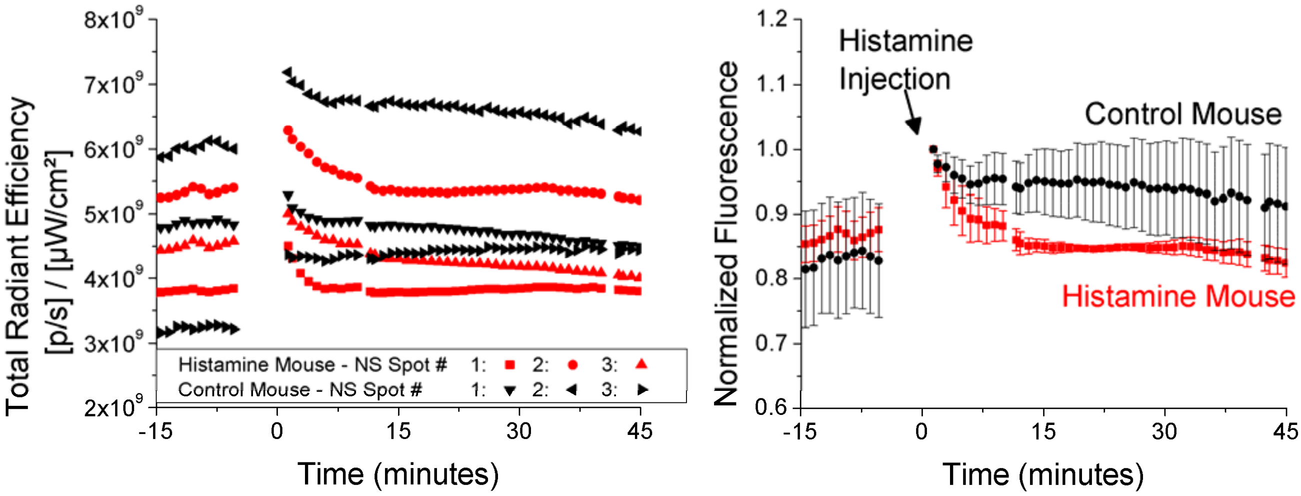

Figure S5. Full dataset for *in vivo* experiments involving i.p. injections of histamine. All show similar trends after injection of histamine. Note that Experiment 4 (lower right) was terminated early due to animal movement during imaging. Experiment 1 (top left) is also Figure 6 in main manuscript.

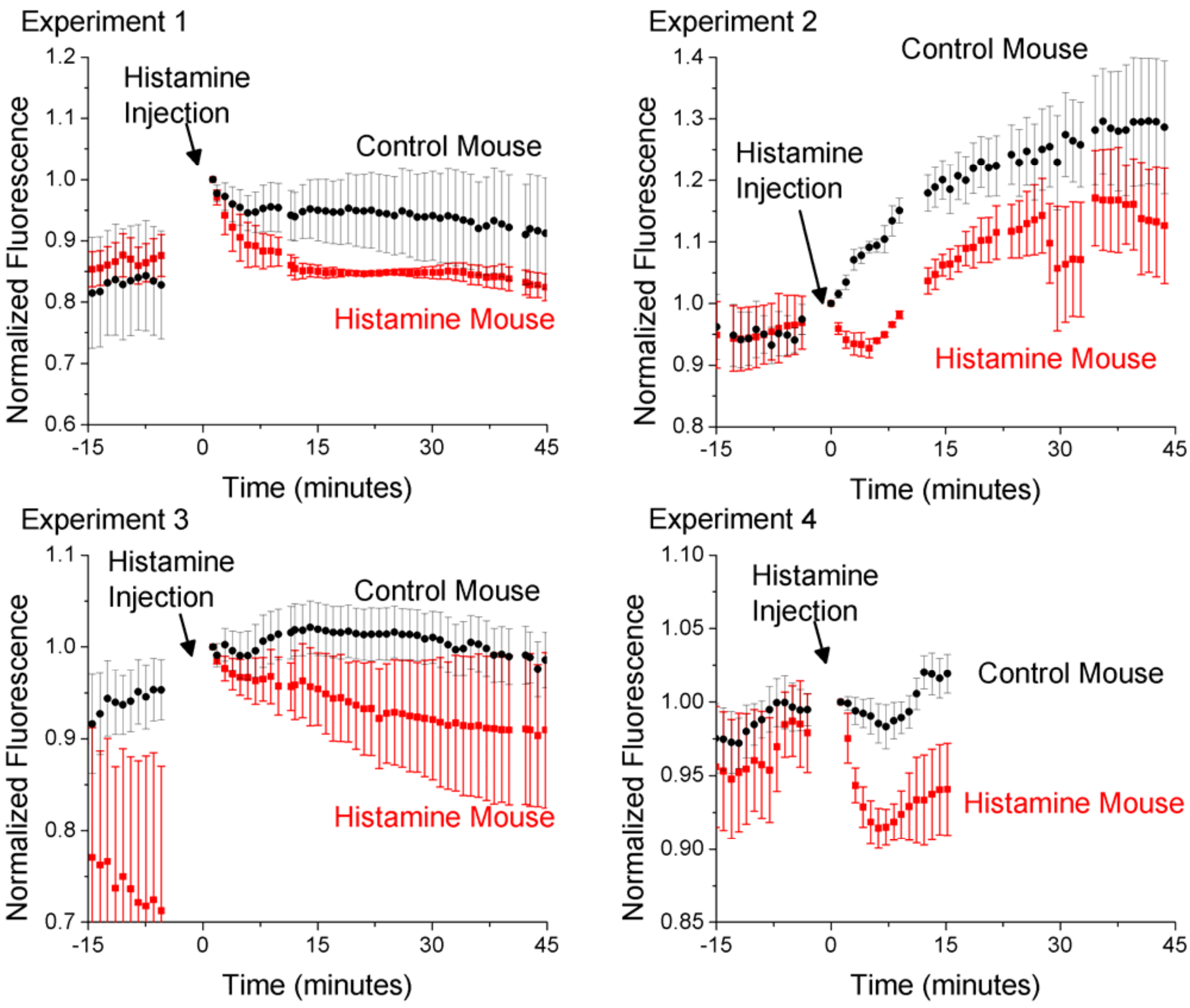

© 2012 by the authors; licensee MDPI, Basel, Switzerland. This article is an open access article distributed under the terms and conditions of the Creative Commons Attribution license (http://creativecommons.org/licenses/by/3.0/).
